# Primers matter: Influence of the primer selection on human fungal detection using high throughput sequencing

**DOI:** 10.1080/19490976.2022.2110638

**Published:** 2022-08-21

**Authors:** Crispin Wiesmann, Konrad Lehr, Juozas Kupcinskas, Ramiro Vilchez-Vargas, Alexander Link

**Affiliations:** a Department of Gastroenterology, Hepatology, and Infectious Diseases, Otto-von-Guericke University Magdeburg, Magdeburg, Germany; b Institute for Digestive Research, Lithuanian University of Health Sciences, Kaunas, Lithuania; c Department of Gastroenterology, Lithuanian University of Health Sciences, Kaunas, Lithuania

**Keywords:** Mycobiome, ITS, 18S, sequencing, fungi

## Abstract

Microbiota research has received an increasing attention for its role in disease development and fungi are considered as one of the key players in the microbial niche. Various sequencing approaches have been applied to uncover the role of fungal community in health and disease; however, little is known on the performance of various primers and comparability between the studies. Motivated by the recent publications, we performed a systematic comparison of the 18S and ITS regions to identify the impact of various primers on the sequencing results. Using four pairs of primers extensively used in literature, fungal community was retrieve from 25 fecal samples, and applying high throughput sequencing; and the results were compared in order to select the most suitable primers for fungal detection in human fecal samples. Considering the high variability between samples, primers described in the Earth microbiome project detected the broadest fungal spectrum suggesting its superior performance in mycobiome research.

During the last years, it has become obvious that microbiota plays a crucial role in health and disease. In the past, the focus when analyzing the human microbiome was mostly on bacteria^1^ and not on other microbes such as fungi;^2^ now there is a growing interest in the fungi inhabiting the human body (mycobiome). The evolving data highlight the importance of fungi as one of the major health issues and they may be associated with and even involved in various diseases, for instance in gastrointestinal diseases,^3,4^ infectious diseases,^5^ autoimmune diseases^6^ and carcinogenesis.^7,8^ However, there are different approaches for targeting fungi such as the 18S rRNA gene or other ITS regions. In a recent work published in Gut microbes, Gosalbes et al. applied the ITS3F and ITS4R targeting the ITS2 region (Internal-transcript-sequence) for interfaction analysis between mycobiome, bacteriome and inflammation in subjects with HIV,^5^ while another paper by Frau et al. utilized 18S rRNA sequencing in interkingdom analysis in Crohn´s disease.^5^ Limited knowledge exists on performance of primers in fungi sequencing. Frequently four popular primer pairs are utilized for mycobiome analysis targeting different regions or genes (Table 1): 18S region, the ITS2 region, the ITS3 region, and the different ITS2 region (labeled as EM): with primers described in the Earth Microbiome Project (https://earthmicrobiome.org/). Even though the gut mycobiome varies from person to person, there are still fungi genera that are found frequently.^9^ These are among others *Saccharomyces* as well as *Candida*, which were found throughout various studies that used the EM,^14^ 18S,^9^ ITS2^4^ or ITS3^9,15^ primer. *Geotrichum* is also an often-found genera in the human gut when using the 18S,^16^ ITS3^15^ or EM^14^ primer. Due to an evolving number of studies, we compared the most frequently used primers for mycobiome analysis focusing on the overall performance and comparison of the data for the three most common fungi genera using a systematic approach.

**Table 1.** List of all used primers and their sequence.

Name	ID Forward	Sequence	ID Rev	Sequence Rev
18S	Euk1152f^9^	(5´- TGAAACTTRAAGRAATTGACGGA- 3´)	Euk1428r^9^	(5´-GGRCATMACDGACCTGYTAT – 3´)
ITS2	ITS2f^10^	(5´- GTGARTCATCGAATCTTT – 3´)	ITS2r^11^	(5´- GATATGCTTAAGTTCAGCGGGT – 3´)
ITS3	ITS3f^12^	(5´- GCATCGATGAAGAACGCAGC – 3´)	ITS4r^12^	(5´- TCCTCCGCTTATTGATATGC – 3´)
EM	ITS1f^13^	(5´- CTTGGTCATTTAGAGGAAGTAA- 3´)	ITS2r^12^	(5´- GCTGCGTTCTTCATCGATGC- 3´)

PCR products from four different pairs of primers were analyzed using the DNA from 25 human fecal samples and yielded a considerably different number of total reads, depending on the paired primers used for fungal detection (Supplementary File 1). For instance, a mean of 6376, 8524, 13475, or 27742 total reads were obtained if the pair of primers 18S, ITS2, ITS3 or EM were used, respectively (Figure 1a). Overall, EM primers retrieved the highest species richness from the samples, detecting a mean of nine species per sample compared with the mean of only four species detected with all the other three pair of primers (Figure 1b). Interestingly, the Simpson index did not detect any differences between all the primers, which might introduce misinterpretations of the data (Figure 1c). Together with the species richness, EM primers were also the primers detecting the highest number of genera, a total of 51 genera compared with approximately 20 genera detected by the other primer pair (Figure 1d). It is worth mentioning that the EM primer can detect the fungal most found in fecal samples, namely *Candida, Geotrichum* and *Saccharomyces* (Figure 1e). Differences between primers were evident when comparing the sequences at genus level. For instance, primers 18S and ITS2 clearly overestimated the abundances of *Saccharomyces* (e.g., in samples S3, S4 or S10 among others), or underestimated the abundance of Geotrichum (e.g., S25 and S28). In summary, EM primers show the ability to detect members of *Candida, Geotrichum, Saccharomyces, Cyberlindnera, Clavispora, Debaryomyces, Pichia, Penicillium* among other fungal genera (figure 1f), thus are more suitable for fungal detection in human fecal samples.

**Figure 1.**
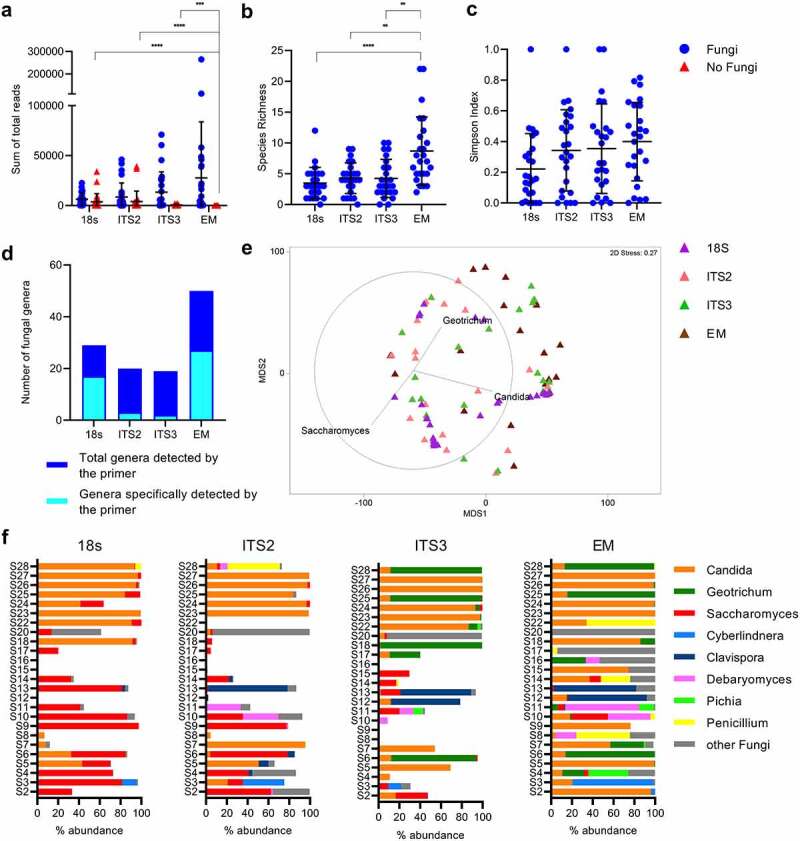
Variation of the sequencing results in fungal analysis depending on the primer used. (a) Average number of fungal and no fungal reads. (b) Species richness and (c) Simpson Index for the different primers. (d) Number of genera found by each primer and the share of these only found by each primer. (e) MDS based on Bray-Curtis resemblance measurement at genus level with vector-overlay representing the most abundant genera. (f) Relative abundance of the most abundant genera for each primer (white share represents non- fungal genera).

The results of this work, where the four most extensively used pairs of primers for assembling the fungal community in literature were studied, are of significance for reporting and analyzing the data. We observed the substantial differences of the outcome of analysis of fungi community that can result in variation of abundance, differences and function depending on the sequencing region. The obtained data shows there is a need for homogenized protocols, validation analysis and standard operative procedure in the scientific community. The reporting standards need to be carefully reviewed to allow retrospective comparison of the published work related to the role of the Kingdom Fungi within the human gastrointestinal tract. With this knowledge, it is possible that the use of a different set of primers, might have provided a more detailed view on the fungal composition as reported for instance by Gosalbes et al.^5^ and Frau et al.^3^

In summary, systematic analysis revealed substantial advantages of EM primers in the assessment of fungal community of the gastrointestinal tract. Due to the observed differences, it is very unlikely that scientific comparison between studies with different fungal primers may be feasible. Metagenomic studies may overcome those limitations; however, the value of EM fungi analysis remains very crucial as a money-saving approach, and the use of the best available primers likely needs to be promoted.

## Material and methods

The present cohort consists of patients’ fecal samples, collected as part of the LiLife Project from the Department of Gastroenterology, Hepatology, and Infectious Disease at the Otto-von-Guericke University Hospital Magdeburg (ID 78/19) and the Twin Registry Center at Lithuanian University of Health Sciences, where samples were taken from 2016 through 2018 (Protocol No: BE-2-10 and P1-52/2005. The study protocols were approved by regional ethics committees and samples were collected following written informed consent and stored at −80°C. From these two cohorts, 25 samples were included in the final analysis. DNA was extracted with the Fast DNA Stool Mini Kit (Qiagen). In the next step four separate PCRs, each with a different primer pair (EM, ITS2 ITS3, and 18S), previously described in the introduction, were conducted. All PCRs were done with 35 cycles, an annealing temperature of 50°C and an elongation time of 45 sec for primers ITS2, ITS3, and 18S and 52°C, and 30 sec and 50°C of annealing temperature for EM primers. After barcoding, sequencing was performed on an Illumina MiSeq (2 × 250 bp, Illumina, Hayward, California, USA). FastQ files were analyzed using the dada2 package,^17^ version 1.10.1, in R. The sequences in the resulting taxonomy table were then manually annotated using the Blast-Algorism and the NCBI database, resulting in a study cohort of 25 samples with 557 sequences, 257 of which belong to the fungi kingdom (Supplementary File 1). The Richness and Simpson Index were calculated in R using the vegan package.^18^ Statistical significands were tested applying the Friedman-Test for a priory defined groups. All p-values were corrected with Benjamini-Hochberg False-Discovery-Rate and q-vales below 0.05 were considered significant. MDS were constructed in Primer 7, underlying a Bray-Curtis resemblance matrix at genus level^19^ and data were visualized in Prism8 (GraphPad Software).

## Supplementary Material

Supplemental Material

## Data Availability

All data are provided in the supplementary file.
